# Serological and molecular diagnostic surveys combined with examining hematological profiles suggests increased levels of infection and hematological response of cattle to babesiosis infections compared to native buffaloes in Egypt

**DOI:** 10.1186/s13071-015-0928-9

**Published:** 2015-06-12

**Authors:** Mona S. Mahmoud, Omnia M. Kandil, Soad M. Nasr, Seham H.M. Hendawy, Salwa M. Habeeb, Dalia M. Mabrouk, Marta G. Silva, Carlos E. Suarez

**Affiliations:** Parasitology and Animal Diseases Department, National Research Center, 33 Bohouth St., Giza, Dokki 12622 Egypt; Cell biology Department, National Research Center, 33 Bohouth St., Giza, Dokki 12622 Egypt; Department of Veterinary Microbiology and Pathology, Washington State University, Pullman, WA USA; Animal Disease Research Unit, Agricultural Research Service, United States Department of Agriculture, Pullman, WA USA

**Keywords:** *B. bigemina*, *B. bovis*, Competitive ELISA, Nested PCR, Semi-nested PCR, Hemogram, Egypt

## Abstract

**Background:**

Babesiosis threatens the development of the cattle and buffaloes industries in Egypt and improved control is needed. The main objectives of this study are surveying the presence of bovine babesiosis in distinct selected bovine and buffalo populations in Egypt using novel molecular and previously validated serological methods, while also comparing the occurrence of hematological alterations among *Babesia* infected cattle and buffalos.

**Methods:**

A total of 253 and 81 blood samples from apparently healthy cattle and buffaloes, respectively, were randomly collected from diverse locations in Egypt. All samples were tested for *Babesia bovis* and *B. bigemina* infection using blood film examination, competitive ELISA (cELISA) and PCR. Novel semi-nested and nested PCR assays for the detection of *B. bovis* and *B. bigemina* respectively, were developed and used to analyze DNA extracted from bovine and buffalo samples. Hematological profiles were studied using a hematological analyzer.

**Results:**

Blood films examination revealed 13.8 % and 7.4 % *Babesia* infection rates in cattle and buffaloes, respectively. However, in cattle, the cELISA detected 32.8 %, 21.3 % and 10.7 % infection rates with *B. bigemina*, *B. bovis* and mixed infection, respectively. In addition, cELISA identified 22.2 %, 22.2 % and 6.2 % infection rates with *B. bigemina*, *B. bovis* and mixed infection, respectively in buffaloes. The semi-nested PCR assay showed that 15 % of the tested samples were positive for *B. bovis* in cattle, but just 3 % in buffaloes. Infections with *B. bigemina* were also found in cattle (32.4 %), but not in buffaloes upon nested PCR analysis. Sequencing analysis confirmed the identity of the PCR amplicons and showed that Egyptian genotypes of *B. bigemina* and *B. bovis* highly resemble sequences previously deposited in GenBank. Hemograms performed on the sampled animals revealed macrocytic hypochromic anemia associated with reduced platelet counts in infected cattle with babesiosis. In addition, marked increases in total leukocyte and granulocytic counts and decreases in lymphocytic counts were found in infected cattle. In contrast, no such hematological anomalies were found in presumably *Babesia-*infected buffaloes.

**Conclusions:**

Frequent occurrence of babesiosis among apparently healthy bovines in Egypt, suggests the need for appropriately designed prevalence studies in this country. Infected bovine, but not buffalo, populations often present hematological disorders compatible with intravascular hemolysis and thrombocytopenia.

## Background

Babesiosis is caused by intraerythrocytic protozoan parasites of the genus *Babesia* that infect a wide range of domestic and wild animals and occasionally humans [1, 2]. Bovine babesiosis is the most significant tick-borne disease of cattle worldwide threatening approximately 1.3 billion animals in vast areas of Asia, Africa, Australia, Central and South America, and Southern Europe [3]. The disease has the potential to cause large economic and sanitary disruptions compromising the livestock industry all over the world [4].

In Egypt, bovine babesiosis is caused mainly by *Babesia bovis* and *Babesia bigemina. B. bovis* parasites are known to be transmitted in this country by *Rhipicephalus microplus* whereas *B. bigemina* is known to be transmitted by both *R. microplus* and *Rhipicephalus annulatus* [5, 6]. Because of its significant economic impact on meat and milk production and on livestock management, it is currently considered as the most important endemic parasitic disease affecting cattle in Egypt [5]. Both *B. bigemina* and *B. bovis* are capable of causing acute disease resulting in life-threatening hemolytic anemia as well as other severe clinical manifestations [7, 8]. Often, especially in bovines younger than a year old, acute disease resolves into persistent disease and the animals become carriers of the parasites, thus ensuring transmission by competent ticks in zoonotic areas. In addition to hemolytic anemia, bovine babesiosis is known to cause other blood disorders including thrombocytopenia [9]. However, there is a paucity of information on the overall impact that these hematological disorders may have in the development of cattle and buffalo industries in Egypt. Assessing hematological disorders can contribute to our better understanding of the pathogenic mechanisms of the disease, but these data can also become a predictor for recovery and wellbeing for the animals in endemic areas, thus this information can be valuable for gauging efficiency upon the application of measures for the control of the disease. Previous studies suggest that there are large numbers of cattle in Egypt infected with subclinical babesiosis [5]. In addition, although clinical evidence suggests that buffaloes are likely more tolerant to *Babesia* infections [10], the hematological response of buffaloes to *Babesia* infection in Egypt remains poorly investigated [10, 11].

Accurate diagnosis of babesial infections plays an important role in monitoring, management and control [12]. A large diversity of diagnostic techniques including blood smear examination, serological tests, and molecular (DNA-based) assays exist currently, yet, each of these methods has limitations. It is increasingly recognized that accurate diagnosis requires a combination of distinct approaches.

Blood smear examination is often considered to be the standard technique for routine diagnosis of babesiosis, particularly in acute cases, and it is considered by some as a gold standard [13]. It is less time consuming than most other methods and is relatively inexpensive [14]. However, the examination of the stained blood smears is insufficient for accurate detection and identification of *B. bovis* and *B. bigemina* during mixed infections and in particular for the detection of carrier cases or sub-clinical infections with low parasitemia [15].

The serological tests, including the indirect fluorescent antibody test (IFAT) and the enzyme-linked immune-sorbent assay (ELISA) are capable of detecting antibodies in carrier animals; therefore they are often used for monitoring surveillance and export certification [16]. Moreover, specific and sensitive competitive ELISA (cELISA) methods have been developed for *B. bovis* and *B. bigemina,* and their use has been validated in several diagnostic laboratories around the world [17–19]. Drawbacks of serological tests include that antibodies can still be detected even years after recovery of infection though no active infection is prevalent, so these methods cannot help in revealing the exact picture of prevalence of infection at that particular point [20]. Nevertheless, the use of serological methods in conjunction with molecular methods aimed at detecting the *B. bovis* and *B. bigemina* DNA in the blood of infected animals can provide a more complete representation of the incidence of bovine babesiosis in endemic areas.

Most of the DNA based methods employed in the diagnosis of *B. bovis* and *B. bigemina* babesiosis are based on standard polymerase chain reaction (PCR) techniques [21, 22]. Appropriate targets for PCR assays include well conserved and species specific genes. PCR techniques permit the identification of parasites at levels far below those identified by the commonly used conventional parasitological techniques for *B. bovis* and *B. bigemina* [20], which is critical for the diagnosis of sub-clinical and latent infection. Consequently, DNA amplification for the diagnostic detection of babesiosis is considered as a powerful tool both in the early phase of infection and in carrier animals. PCR results for *Babesia sp*. can be rapidly made available and as few as three parasites in 100 μl of blood can yield a positive result [23]. Additionally, the sensitivity of a PCR assay can be increased several fold by performing a semi-nested (snPCR) or nested PCR (nPCR), where two different sets of amplification primers are used [2]. This feature not only improves the sensitivity of the assay, but also confirms the specificity of the first round product [24].

The aim of the present study is to estimate the presence of bovine and buffaloes babesiosis in distinct populations in Egypt using blood smear examination, competitive inhibition ELISA and novel PCR procedures based on the amplification of the recently identified and highly conserved rhoptry associated protein (*rap)-1c* gene of *B. bigemina* [25] and rap-1 related antigen (*rra)* gene of *B. bovis* [26]. We also compared *Babesia*-associated hematological alterations in the cattle and buffalo populations sampled in this study.

## Methods

### Collection and processing of blood samples

Blood samples were collected from clinically healthy 253 cattle and 81 buffaloes examined for diagnosis of babesiosis from Kafr El-Sheikh, El-Behaira and Cairo governorate. The blood collected without anti-coagulants were processed for serum. Sera were stored in -20 °C until used in serological tests. The blood collected with anti-coagulant EDTA (ethylenediaminetetraacetic acid tripotassium) was used for PCR and hematology. For PCR, blood was washed three times with PBS pH 7.2 and stored at -20 °C. Blood from *B. bigemina* experimentally infected animals used in the PCR comparative studies was kindly provided by Dr. Massaro Ueti (ADRU-USDA), and the *B. bovis* Mo7 experimentally infected samples were derived from a previously published experiment [27]. The protocol of infection and all animal handling, was approved by the Institutional Animal Care and Use Committee, IACUC, Washington State University (#03735-008 approved on 12/9/2009).

### Analysis of samples

#### Blood film examination

Blood smears stained with DIFF-3 Rapid Staining kit were prepared from all animals included in this study and examined by light microscopy at 1000x magnification.

#### Competitive ELISA

The *B. bovis* and *B. bigemina* Antibody Test Kits (cELISA) were kindly donated by VMRD Inc. (Pullman, WA, USA). Optical density (O.D.) values were determined at 650 nm using EL_X_ 800 Universal microplate reader Bio-TEK instruments, INC, USA. The results were expressed as a value of the percent inhibition (%I) according to the following formula:

$$ \left(\%\mathrm{I}\right):\;\%\mathrm{I}=100\hbox{-} \left[\left( sample\;O.D.\times 100\right)/\left( mean\; negative\; control\;O.D.\right)\right] $$

Samples were classified as positive if the %I value was above 40 % and negative if the %I value was less than 40 %. These cut-off values were established by the manufacturer of the kit.

#### Extraction of DNA

The DNA was extracted from the blood samples according to the instructions of the manufacturer using FTA® Elute cards, Whatman Cat. No.WB120410.

#### Novel PCR methods for the molecular diagnostic of bovine and buffalo babesiosis

Selection of the *B. bovis rra* and *B. bigemina rap-1c* genes as targets for the development of novel PCR molecular diagnostic tools for the detection of bovine babesiosis was based on their high degree of species-specific conservation among distinct strains, and their limited levels of expression in merozoites [25, 26]. Semi-nested and nested PCR techniques were developed to amplify regions of the *rra* gene [26] (GenBank accession number: XM_001610900) from *B. bovis* DNA and the *rap-1c* gene [25] (GenBank Accession number: AY146983.1) from *B. bigemina* DNA. Sequences derived from the *rra* (bp 114-937) and *rap-1c* (bp 1-602) genes were amplified from *B. bovis* and *B. bigemina* genomic DNA using the set of primers described in Table 1, and cloned into pCR™ 2.1-TOPO® (Life technologies) plasmids. Taking into consideration the size of each resulting plasmid, it was estimated that 1 ng of *B. bovis rra* 2.1-TOPO plasmid contains 2.1 × 10^8^ molecules of the target gene and 1 ng of *B. bigemina rap-1c* 2.1-TOPO plasmid contains 2.2 × 10^8^ molecules of the target gene. To estimate the sensitivity of the assays, we performed serial dilutions of known amounts of plasmid that we then amplified by PCR using the protocol described below. The presence and size of the final PCR products was determined in 1.5 % agarose gels and stained with SYBR Safe (Invitrogen). All PCR amplicons were sequenced for confirming specificity. PCR reactions were performed in a final volume of 25 μl containing (12.5 μl JumpStart RED *Taq* Ready Mix PCR reaction mix (Sigma- Aldrich), 10 pmol of each primer and 9.5 μl water). All primers were synthesized by Integrated DNA technologies Inc. (California, USA). 2 μl of template DNA was used for the primary PCR. The snPCR and nPCR utilized 0.5 μl of primary PCR product as template.

**Table 1:** Sequences of the oligonucleotide primers used in the PCR assays and expected PCR product sizes

Parasite	Primer name	Sequence	Product Size	Source gene name and reference
*Babesia bovis*	BoF	5’ ATTGGCATCTGGGCTAAGTG 3’	823 bp	*B. bovis* Rhoptry associated protein related antigen (*rra*) gene
	BoR	5’ CAGCCCATTTCACAGGTTTT 3’		
	BoNF	5’ TGTTCCTGAGCCGCTATCTT 3’	387 bp	
	BoNR	5’ CAGCCCATTTCACAGGTTTT 3’		
*Babesia bigemina*	BiF	5’ ATGATTCACTACGCTTGCCTC 3’	600 bp	*B. bigemina* Rhoptry associated protein (*rap-1c*) gene
	BiR	5’ GTCTTGTAGTATATGGCGGTCATGTAG 3’		
	BiNF	5’ TCTCGAAGACAGCGAACAGA 3’	236 bp	
	BiNR	5’ GTGAAGCTGGTAGGGGTCAG 3’		

The primer sets used for the primary reaction were: BoF and BoR for the amplification of the *rra B. bovis* gene, and BiF and BiR for the amplification of the *rap-1c B. bigemina* gene. The primer sets used for semi-nested / nested PCR reaction were: BoNF and BoNR for amplification of the *rra* gene of *B. bovis* and BiNF and BiNR amplification of the *rap-1c* gene of *B. bigemina*

The thermo-cycling conditions used for the *B. bovis* primary PCR were: 95 °C for 3 min followed by 25 cycles, each consisting of denaturation at 94 °C for 30 sec, annealing at 60 °C for 1 min, and extension at 72 °C for 1 min. The program also included a final extension step at 72 °C for 5 min. For the semi-nested PCR the same conditions were used except the number of cycles used was 35.

The thermocycling conditions used for the *B. bigemina* primary PCR consisted of: 95 °C for 3 min followed by 25 cycles, each consisting of denaturation at 95 °C for 30 sec, annealing at 61.2 °C for 30 sec, and extension at 72 °C for 30 sec, followed by a final extension step at 72 °C for 5 min. The thermocycling conditions used for the *B. bigemina* nPCR were: 95 °C for 3 min followed by 35 cycles, each consisting of denaturation at 95 °C for 30 sec, annealing at 63.1 °C for 30 sec, and extension at 72 °C for 30 sec, followed by a final extension step at 72 °C for 5 min.

The *B. bovis* and *B. bigemina* genomic DNA derived from cultured T3Bo *B. bovis* and Puerto Rico *B. bigemina* strains respectively used as positive controls were provided by the Animal Disease Research Unit, Agricultural Research Service, USDA, WSU, Pullman. A negative control with no DNA template was always included for PCR amplification. Bovine DNA extracted from blood from non-infected calves was also used as a control for the nested and semi-nested PCRs. Amplified DNA samples were electrophoresed on 1.5 % agarose gels and stained with SYBR Safe. The length of the amplified products was estimated using a 100 base pair (bp) DNA ladder and the amplified products were visualized with an UV trans-illuminator (Bachofer D7410) and photographed using gel Documentation system (BioDocAnalyze-Biometra Analytic GmbH). Amplicons presenting visible unique bands of approximately 387 base pairs (bp) and 236 bp in gel electrophoresis analysis were considered positive for *B. bovis rra* and the *B. bigemina rap-1c* genes, respectively. The relative sensitivities of the *B. bovis* and *B. bigemina* nested and semi-nested-PCR reactions of the tests based on the *B. bovis rra* and the *B. bigemina rap-1c* genes were compared with standardized nested PCR tests described previously [28]. The two new PCR methods were initially tested on gDNA extracted from *B. bovis* and *B. bigemina in vitro* cultures and from *B. bovis* and *B. bigemina* experimentally infected calves in order to compare their performance with previously standardized nested PCR methods for the detection of *B. bovis* and *B. bigemina* in bovine samples by Figueroa *et al.* 1993 [28]. The previously reported nested PCR for *B. bovis*, based on the *rap-1* gene as the target of amplification [28] was compared with a semi-nested PCR targeting the *B. bovis rra* gene, using serial dilutions of genomic DNA extracted from *in vitro* cultured *B. bovis* parasites from the T3Bo strain. Similar comparative analysis was performed for the novel *rap-1c* gene based nested PCR for the detection of *B. bigemina*.

#### Sequencing of PCR products

The PCR products were purified for sequencing using the QIA quick Spin PCR Purification kit (Qiagen, Courtaboeuf, France) according to the manufacturer’s instructions. Sequencing reactions were performed using oligonucleotide primers that were used in the PCR (Table 1). Sequencing of the PCR products was performed by the GATC Company with an ABI 3730xl DNA sequencer, using the nested forward and reverse primers. Each sequencing reaction was repeated three times in both the forward and reverse directions before being accepted for analysis. Sequences derived from *Babesia* sp. were assembled using ChromasPro 1.49 beta (Technelysium Pty. Ltd., Tewantin, QLD, Australia).

#### Hematological investigations

Complete blood analysis was performed on 133 blood samples (107 cattle and 26 buffaloes) from the three Egyptian governorates using a hematological analyzer (MEDONIC CA620, Sweden). The criteria for the selection of the blood samples for hematological examinations were based on: breed consistency; cross-bred cattle and water buffaloes; gender (all animals studied are male); and age range (all animals tested are between 2-3 years old). The analysis included erythrogram consisted of red blood cell count (RBC), hematocrit (HCT), hemoglobin (Hb) concentration and red cell indices, mean corpuscular volume (MCV), mean corpuscular hemoglobin (MCH), mean corpuscular hemoglobin concentration (MCHC), red blood cell distribution width absolute (RDWA), red cell distribution width percentage (RDW%), leukogram (including white blood cell count (WBC), lymphocytes, granulocytes and mid cells (MID) and platelet count (PLT)), mean platelet volume (MPV), platelet distribution width (PDW), plateletcrit (PCT), and large platelet concentration ratio (LPCR).

#### Statistical analysis

All data were subjected to statistical analysis including the calculation of the mean and standard error. Differences between infected groups detected by different techniques and non-infected groups were tested for significance using a one-way analysis of variance followed by Duncan’s multiple range test. Differences were considered significant at *P* < 0.05 level [29] using SPSS software version 15.

## Results

### Diagnosis of bovine babesiosis using direct blood film microscopic examination

We examined a total of 253 cattle and 81 buffaloes’ microscopic stained slide samples. Because of the difficulties in objectively providing differential diagnosis among *B. bovis* and *B. bigemina* parasites using this method, added to the possibility of frequent double *B. bovis* and *B. bigemina* infections in bovines, we decided to report our microscopic examination findings as “*Babesia* sp.” Direct microscopic examination resulted in the detection of *Babesia* infected erythrocytes in 13.8 % and 7.4 % samples of cattle and buffaloes respectively.

### Serological diagnosis of bovine and buffalo babesiosis using cELISA

We performed cELISA tests for the detection of *B. bovis* and *B. bigemina* antibodies on serum samples obtained from the 253 bovines and 81 buffaloes as described above. The cELISA data performed on cattle sera (Table 2) revealed 32.8 %, 21.3 % and 10.3 % infection with *B. bigemina*, *B. bovis* and mixed infection, respectively. The cELISA test performed on buffaloes sera (Table 3), detected 22.2 %, 22.2 % and 6.2 % infection with *B. bigemina*, *B. bovis* and mixed infection, respectively.

**Table 2:** Results of cELISA and PCR tests for the diagnosis of *B. bovis* and *B. bigemina* performed on sera from cattle from Kafr El-Sheikh, El-Beheira and Cairo governorates in Egypt

		ELISA						PCR					
		*B. bigemina*		*B. bovis*		*B. bigemina and B. bovis*		*B. bigemina*		*B. bovis*		*B. bigemina and B. bovis*	
Governorate	No. tested animals	No. of positive animals	Infection (%)	No. of positive animals	Infection (%)	No. of positive animals	Co-infection (%)	No. of positive animals	Infection (%)	No. of infected animals	Infection (%)	No. of infected animals	Co-infection (%)
Kafr El-Sheikh	147	47	31.9	38	25.9	18	12.2	64	43.5	33	22.4	17	11.6
El-Beheira	88	31	35.2	10	11.4	3	3.4	0	0	2	2.3	0	0
Cairo	18	5	27.8	6	33.3	5	27.8	18	100	3	16.7	5	27.8
Total	253	83	32.8	54	21.3	26	10.3	82	32.4	38	15.0	22	8.7

**Table 3:** Results of cELISA and PCR tests for the diagnosis of *B. bovis* and *B. bigemina* performed on sera from buffalo sera from Kafr El-Sheikh and El-Beheira governorates in Egypt

		ELISA						PCR			
		*B. bigemina*		*B. bovis*		*B. bigemina and B. bovis*		*B. bigemina*		*B. bovis*	
Governorate	No. tested animals	No. of positive animals	Infection (%)	No. of positive animals	Infection (%)	No. of positive animals	Co-infection (%)	No. of positive animals	Infection (%)	No. of infected animals	Infection (%)
Kafr El-Sheikh	7	5	71.4	3	42.9	2	28.6	0	0	2	28.6
El-Beheira	74	13	17.6	15	20.3	3	4.1	0	0	1	1.4
Total	81	18	22.2	18	22.2	5	6.2	0	0	3	3.7

### Molecular detection of *B. bovis* and *B. bigemina* in infected bovines and buffaloes using PCR

**Fig. 1:**
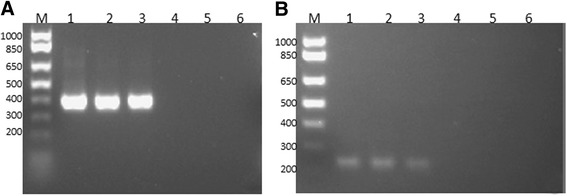
Agarose gel electrophoresis analysis: Serial dilutions of known amounts of purified plasmid DNA amplified by PCR. Molecular size ladder in base pair (bp), 100 bp DNA ladder, (lane M). **a**. Plasmid pCR™ 2.1-TOPO-*B. bovis rra* amplified using snPCR. The amounts of plasmid amplified are as follows: 10 femtogram (fg) (lane 1), 1 fg (lane 2), 0.1 fg (lane 3), 0.01 fg (lane 4), 0.001 fg (lane 5), negative control, no plasmid (lane 6). **b**. Plasmid pCR™ 2.1-TOPO-*B. bigemina rap-1c* amplified using nPCR. The amounts of plasmid amplified are as follows: 10 fg (lane 1), 1 fg (lane 2), 0.1 fg (lane 3), 0.01 fg (lane 4), 0.001 fg (lane 5), negative control, no plasmid (lane 6)

**Fig. 2:**
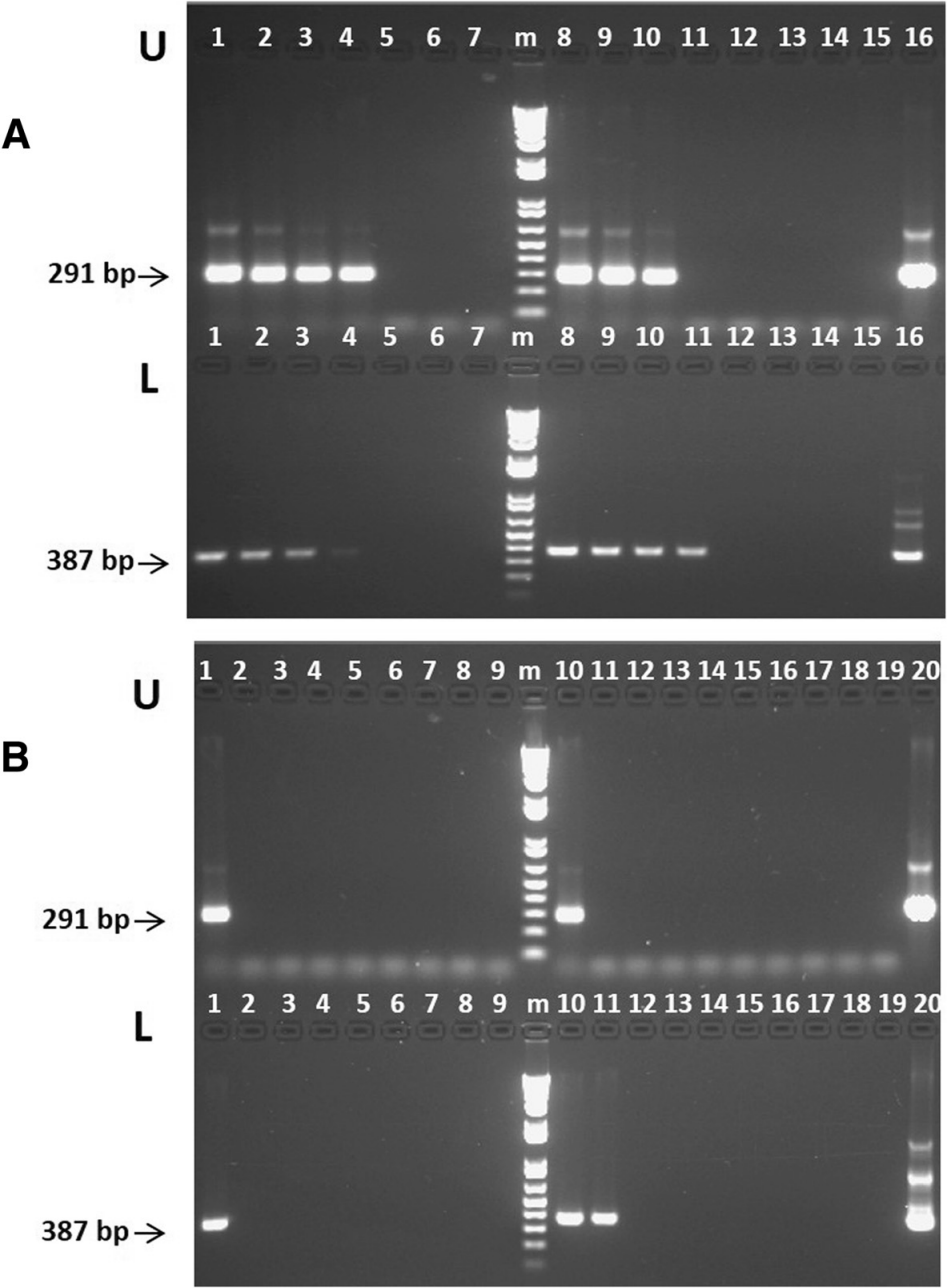
Agarose gel electrophoresis separation of PCR products of the *B. bovis* PCR detection methods: Two replica comparisons of the relative sensitivity of the nested PCR and semi-nested PCR methods for the detection of *B. bovis* DNA using genomic DNA extracted from *in vitro* cultured parasites from the *B. bovis* T3B strain (A) and from experimentally infected bovine (Mo7 strain) (B). **a**. The two PCR methods used were based on the *B. bovis rap-1* (U) and on the *rra* (L) genes. The amounts of DNA amplified were 5 ng (lanes 1,8); 5×10^−1^ ng (lanes 2,9); 5×10^−2^ ng (lanes 3,10); 5×10^−3^ ng (lanes 4,11); 5×10^−4^ ng (lanes 5,12); 5×10^−5^ ng (lanes 6,13); 5×10^−6^ ng (lanes 7,14). Lane 15 represents PCR amplifications without adding DNA, and lane 16 represents PCR amplifications using plasmid *pCR™ 2.1-TOPO B. bovis-rap-1* (U) or plasmid *pCR™ 2.1-TOPO B. bovis-rra-1* (L) as positive controls. **b**. The two PCR methods used were based on the *B. bovis rap-1* (U) and on the *rra* (L) genes. The amounts of DNA amplified were 5 ng (lanes 1,10); 5×10^−1^ ng (lanes 2,11); 5×10^−2^ ng (lanes 3,12); 5×10^−3^ ng (lanes 4,13); 5×10^−4^ ng (lanes 5,14); 5×10^−5^ ng (lanes 6,15); 5×10^−6^ ng (lanes 7,16); 5×10^−7^ ng (lanes 8,17); and 5×10^−8^ ng (lanes 9,18). Lane 19 represents PCR amplification without adding DNA, and lane 20 represents PCR amplifications using plasmid *pCR™ 2.1-TOPO B. bovis-rap-1* (U) or plasmid *pCR™ 2.1-TOPO B. bovis-rra-1* (L) as positive controls. Molecular size ladder in base pair (bp), 100 bp DNA ladder (lane m). The amplicons of interest are indicated at the left

We first determined the optimal conditions and sensitivity of nested and semi-nested PCR reactions for the detection of *B. bigemina* and *B. bovis*, respectively. When these two methods were compared using plasmid DNA encoding for the target genes, both reactions have similar levels of sensitivity, and are able to clearly detect down to 0.1 fg for the *B. bigemina rap-1c* and *B. bovis rra* genes (Fig. 1a and b) using nPCR and snPCR respectively. In addition, the performances of the novel *rra* based semi-nested PCR and rap-1c tests were compared with previously established *B. bovis rap-1*- based nPCR and *B. bigemina* nPCR tests respectively, before applying them for the analysis of the field samples. Results on the application of the *rap-1* nested PCR on genomic DNA (gDNA) from *in vitro* cultured parasites are shown in the upper panel of Fig. 2a, and results for the *rra* semi-nested PCR are shown the in the lower panel. Both tests are capable of producing PCR products reliably down to at least 5×10^−2^ ng of gDNA, and so, they appear to have comparable sensitivity. Importantly, the *rra-*based PCR method generates single PCR final products upon gel analysis. When similar comparisons were performed using 10 fold serial dilutions of gDNA extracted from persistently and experimentally *B. bovis* Mo7 strain infected bovines with unknown parasitemia (less than 0.5 % PPE) (Fig. 2b), both tests again appear to perform similarly and to have a comparable level for detection of *B. bovis* DNA. In addition, sequencing of the PCR products of 291 bp (*rap-1* targeted PCR) and 387 bp (*rra* targeted PCR) demonstrated the specificity of the amplicons as true *rap-1* and *rra* sequences. Importantly, none of the tests amplify bovine gDNA (data not shown), the sequences of the *rra* gene were previously shown to be identical among the T3Bo and the Mo7 strains [26], and sequencing demonstrates that the novel PCRs only amplify their expected target genes. Taking together the comparisons of both PCR tests performed on DNA extracted from *in vitro* grown cultures and *in vivo B. bovis* infected calves, suggests that both tests have similar performances in terms of sensitivity and specificity. However, the *rra*-based test seems to have a slight advantage in terms of specificity since it invariably generated a single product upon PCR.

**Fig. 3:**
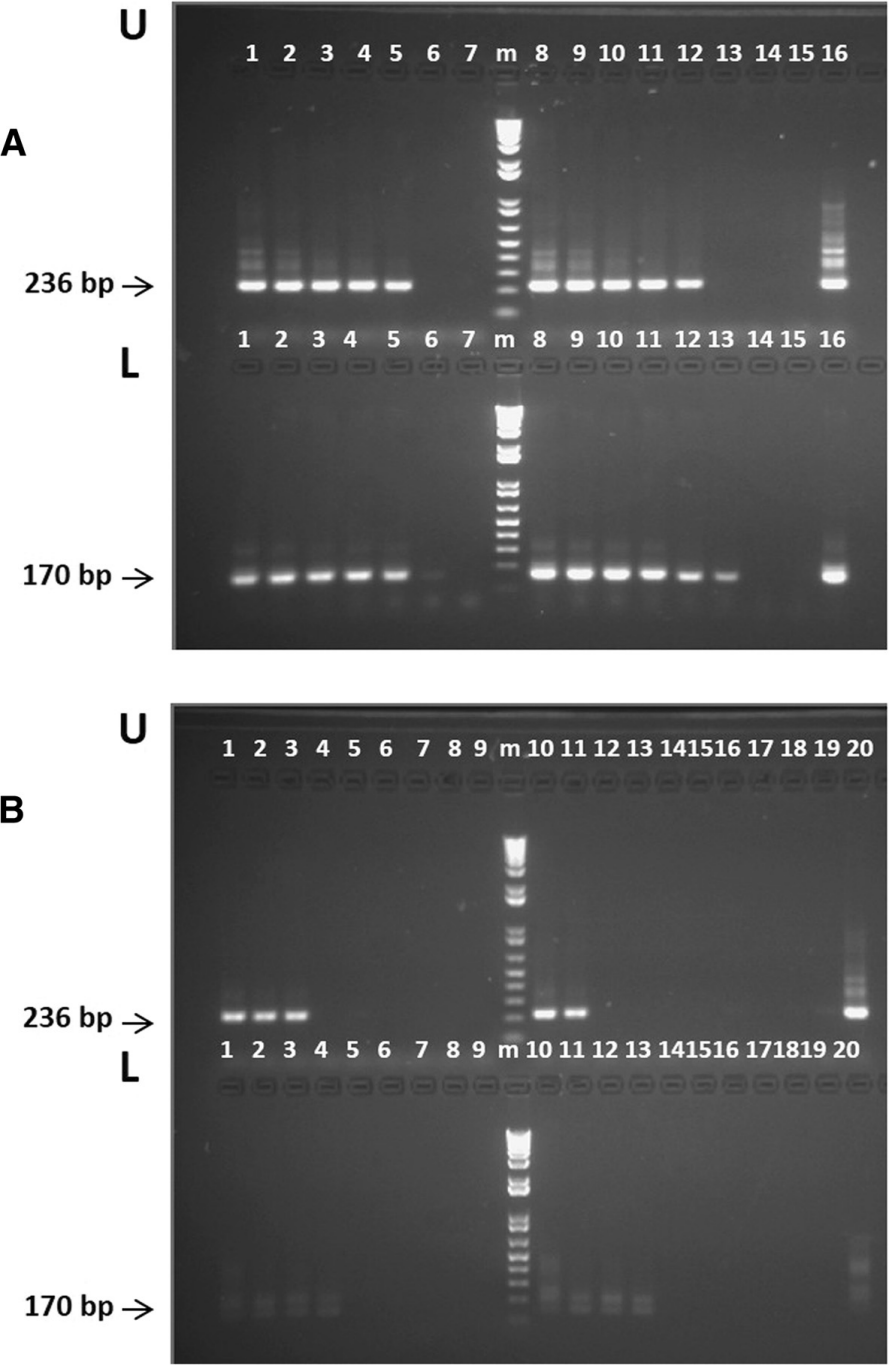
Agarose gel electrophoresis separation of PCR products of the *Babesia bigemina* PCR detection methods: Two replica comparisons of the relative sensitivity of the nested PCR and semi-nested PCR methods for the detection of *B. bigemina* DNA using genomic DNA extracted from *in vitro* cultured parasites from the *B. bigemina* Puerto Rico strain (A) and from an experimentally infected bovine with the *B. bigemina* Puerto Rico strain (B). **a**. The two PCR methods used were based on the amplification of a *B. bigemina* specific (U) and on the *rap-1c* (L) genes. The amounts of DNA amplified were 5 ng (lanes 1 and 8); 5×10^−1^ ng (lanes 2,8); 5×10^−2^ ng (lanes 3,9); 5×10^−3^ ng (lanes 4,10); 5×10^−4^ ng (lanes 5, 11); 5×10^−5^ ng (lanes 6,12); and 5×10^−6^ ng (lanes 7,13); lane 14 represents PCR amplifications without adding DNA, and lane 16 represents PCR amplifications using plasmid *pCR™ 2.1-TOPO B. bigemina-1* (U) or plasmid *pCR™ 2.1-TOPO B. bigemina-rap-1c* (L) as positive controls. **b**. The two PCR methods used were based on the *B. bigemina* gene (U) and on the *rap-1c* (L) genes. The amounts of DNA amplified were 5 ng (lanes 1,10); 5x10^−1^ ng (lanes 2,11); 5×10^−2^ ng (lanes 3,12); 5×10^−3^ ng (lanes 4,13); 5×10^−4^ ng (lanes 5,14); 5×10^−5^ ng (lanes 6,15); 5×10^−6^ ng (lanes 7,16); 5×10^−7^ ng (lanes 8,17); and 5×10^−8^ ng (lanes 9, 18). Lane 19 represents PCR amplification without adding DNA, and lane 20 represents PCR amplifications using plasmid *pCR™ 2.1-TOPO B. bigemina* (U) or plasmid *pCR™ 2.1-TOPO B. bigemina-rap-1* (L) as positive controls. Molecular size ladder in base pair (bp), 100 bp DNA ladder (lane m). The amplicons of interest are indicated at the left

The data shown in the upper panel of the gel in Fig. 3a depicts nested PCR amplification using the *rap-1c* primer sets performed on 5 ng of *in vitro* cultured *B. bigemina* gDNA, where lanes 2-9 represent identical PCR amplifications performed on serial 10-fold dilutions from this material. The lower panel of the gel shows the data obtained from nested PCR amplifications performed on identical ten-fold dilutions *in vitro* cultured infected erythrocytes, but using the previously described by Figueroa *et al* primer set [28]. All experiments were performed in duplicate. Overall, the data suggests that both PCR methods have similar limits for the detection of *B. bigemina* in *in vitro* cultured parasites, consistently detecting up to 5x10^−4^ ng of gDNA. We also compared the performance of these two *B. bigemina* nPCR amplification methods on an identical set of target genomic DNAs extracted from a *B. bigemina* infected bovine with an unknown parasitemia. None of these PCR methods generated PCR products when used on purified bovine gDNA and sequencing of the nPCR products of 236 and 170 bps confirmed specificity of the assays. However, the data in Fig. 3b shows that the PCR products generated using the *B. bigemina rap-1c* nPCR primer set on the DNA extracted from the experimentally infected animals were better visualized than the previously published *B. bigemina* primer set [28]. Overall, the two PCR procedures tested for the amplification of *B. bigemina* DNA showed similar levels of sensitivity and specificity. Yet, because better PCR product visualization in gels was obtained using the *rap-1c*-based PCR method, we decided to use this method for the molecular detection of *B. bigemina* field samples derived from cattle from distinct areas in Egypt, as described below.

The *rap-1c B. bigemina* and *rra B. bovis* based PCR methods described above were used to detect the presence of *B. bigemina* and *B. bovis* on total genomic DNA blood samples of cattle (n = 253) and buffaloes (n = 81). Fifteen percent of the cattle samples and 3.7 % of the buffaloes samples tested were found positive for *B. bovis* using snPCR. The percent of infection with *B. bigemina* calculated in cattle using nPCR was 32.4 %, whereas none of the buffalo samples were positive in nPCR for *B. bigemina*. In addition, mixed infection with *B. bigemina* and *B. bovis* was found in 8.7 % of cattle samples (Tables 2 and 3).

Sequencing of the 236 nucleotides long nPCR amplicons originating from the samples positive for *B. bigemina* revealed 100 % identity with the expected *B. bigemina rap-1c* gene of the strain S1A. In addition, the 283 nucleotide snPCR products positive for *B. bovis* were also 100 % identical to the sequence in the *B. bovis rra* gene. Sequences originating from the Egyptian positive samples were deposited in GenBank with accession number: KM212998 for *B. bigemina*, and KM213000 for *B. bovis*.

### Hematological findings

The data for the hematological analysis performed on control non-infected and naturally *Babesia sp-*infected cattle and buffaloes detected by different techniques are illustrated in Tables 4 and 5.

**Table 4:** Hematological profiles in non-infected and *Babesia* sp infected, cattle as detected by different methods

Groups Parameters	Control Non-infected	Method for the detection of *Babesia* sp. used in cattle				Sig.
		Blood film	cELISA	nPCR	Blood film + cELISA + nPCR	
No. of Animals	23	5	28	43	8	
Red blood cell count (×10^6^/μl)	7.09 ± 0.18^a^	6.03 ± 0.10^bc^	6.31 ± 0.14^b^	5.45 ± 0.14^c^	6.13 ± 0.19^bc^	***
Hematocrit (%)	27.73 ± 0.32^a^	25.34 ± 0.41^ab^	25.58 ± 0.44^ab^	24.10 ± 0.58^b^	23.90 ± 0.96^b^	***
Hemoglobin (g/dl)	11.16 ± 0.23^a^	9.14 ± 0.49^b^	9.00 ± 0.13^b^	8.42 ± 0.19^b^	8.48 ± 0.32^b^	***
Mean corpuscular volume (fl)	39.53 ± 0.86^b^	42.08 ± 0.95^ab^	40.87 ± 0.78^b^	44.43 ± 0.60^a^	38.95 ± 0.76^b^	***
Mean corpuscular hemoglobin (pg)	15.86 ± 0.35^a^	15.19 ± 0.91^ab^	14.40 ± 0.27^bc^	15.53 ± 0.19^ab^	13.82 ± 0.29^c^	***
MCHC (mg/dl)	40.20 ± 0.58^a^	36.03 ± 1.59^b^	35.26 ± 0.21^b^	34.97 ± 0.09^b^	35.48 ± 0.21^b^	***
Red blood cell distribution width (fl)	31.51 ± 0.73^b^	43.74 ± 5.00^a^	31.38 ± 1.14^b^	33.54 ± 0.69^b^	31.66 ± 0.73^b^	***
Red blood cell distribution width (%)	21.01 ± 0.42^b^	26.96 ± 2.35^a^	19.44 ± 0.30^bc^	18.20 ± 0.30^bc^	19.93 ± 0.36^bc^	***
White blood cell count (×10^3^/μl)	10.93 ± 0.40^bc^	13.48 ± 0.37^a^	12.20 ± 0.70^ab^	9.63 ± 0.39^c^	11.56 ± 0.21^abc^	**
Lymphocytes (×10^3^/μl)	5.6 ± 0.28^a^	6.73 ± 0.54^a^	5.58 ± 0.39^a^	3.51 ± 0.29^b^	3.72 ± 0.71^b^	***
Lymphocytes (%)	52.38 ± 1.20^a^	50.44 ± 5.05^a^	45.77 ± 1.80^ab^	37.20 ± 2.53^bc^	32.38 ± 6.29^c^	***
Granulocytes (×10^3^/μl)	4.08 ± 0.14^b^	5.60 ± 0.94^ab^	5.52 ± 0.39^ab^	5.26 ± 0.38^b^	6.90 ± 0.77^a^	*
Granulocytes (%)	37.89 ± 1.34^c^	40.96 ± 5.46^c^	44.90 ± 1.76^bc^	53.63 ± 2.61^ab^	59.49 ± 6.42^a^	**
MID cells (×10^3^/μl)	1.08 ± 0.07^a^	1.15 ± 0.06^a^	1.10 ± 0.06^a^	0.86 ± 0.03^b^	0.94 ± 0.05^a^	*
MID cells (%)	9.73 ± 0.30	8.60 ± 0.55	9.33 ± 0.46	9.17 ± 0.31	8.14 ± 0.45	NS
Platelet count (×10^3^/μl)	269.78 ± 14.93^a^	232.00 ± 2.86^b^	214.75 ± 16.22^bc^	158.16 ± 8.40^c^	224.63 ± 29.66^b^	***
Mean platelet volume (fl)	6.47 ± 0.07^a^	6.30 ± 0.09^abc^	6.44 ± 0.07^ab^	6.17 ± 0.04^bc^	6.10 ± 0.09^c^	***
Platelet distribution width (fl)	10.43 ± 0.12^a^	10.14 ± 0.18^ab^	10.44 ± 0.12^a^	10.00 ± 0.08^ab^	9.78 ± 0.12^b^	**
Plateletcrit (%)	0.18 ± 0.01^a^	0.16 ± 0.00^a^	0.14 ± 0.01^a^	0.10 ± 0.01^b^	0.17 ± 0.02^a^	**

Hemogram values expressed as mean ± SE (Standard error) for each technique. Means followed by different superscripts (^a^, ^b^, ^c^) within the same row are significantly different at (*P* < 0.05). Two means that are not follow by the same letter (a, b, or c) are significantly different (*P* < 0.05). Two means followed with the same letter implies that they are not significantly different. Sig.: represents statistical significance. * *P* < 0.05, ** *P* <0.01, *** *P* <0.001**.** NS = non- significant. MCHC = mean corpuscular hemoglobin concentration

**Table 5:** Hematological profiles in non-infected and *Babesia* sp infected buffaloes as detected by different methods

Groups Parameters	Control Non-infected	Method for the detection of *Babesia* sp. used in buffaloes			Sig.
		Blood film	cELISA	Blood smears + cELISA + nPCR	
No. of Animals	10	4	10	2	
Red blood cell count (×10^6^/μl)	7.01 ± 0.38	6.49 ± 0.62	6.70 ± 0.38	7.88 ± 0.24	NS
Hematocrit (%)	34.04 ± 1.48	31.78 ± 0.88	33.22 ± 1.53	40.45 ± 4.55	NS
Hemoglobin (g/dl)	11.32 ± 0.43	10.50 ± 0.40	10.89 ± 0.50	13.20 ± 1.00	NS
Mean corpuscular volume (fl)	48.81 ± 0.74	49.75 ± 2.85	50.13 ± 1.75	51.25 ± 4.25	NS
Mean corpuscular hemoglobin (pg)	16.29 ± 0.35	16.40 ± 0.80	16.42 ± 0.49	16.75 ± 0.75	NS
MCHC (mg/dl)	33.40 ± 0.34	32.98 ± 0.38	32.85 ± 0.27	32.85 ± 1.25	NS
Red blood cell distribution width (fl)	20.11 ± 0.43	17.58 ± 0.38	19.53 ± 0.77	20.50 ± 1.50	NS
Red blood cell distribution width (%)	42.22 ± 0.74	40.65 ± 2.65	42.96 ± 1.60	45.40 ± 3.30	NS
White blood cell count(×10^3^/μl)	8.74 ± 0.30	7.20 ± 2.94	7.30 ± 0.43	7.00 ± 0.70	NS
Lymphocytes (×10^3^/μl)	2.14 ± 0.22	1.70 ± 0.58	2.34 ± 0.56	1.80 ± 0.40	NS
Lymphocytes (%)	24.85 ± 2.32	28.45 ± 3.84	30.95 ± 5.49	25.90 ± 3.00	NS
Granulocytes (×10^3^/μl)	5.92 ± 0.28	4.85 ± 2.17	4.00 ± 0.37	4.35 ± 0.45	NS
Granulocytes (%)	67.94 ± 2.32	62.90 ± 4.10	56.60 ± 4.93	61.75 ± 0.45	NS
Mid cells (×10^3^/μl)	0.68 ± 0.03	0.65 ± 0.20	0.96 ± 0.08	0.85 ± 0.15	NS
Mid cells (%)	7.21 ± 0.16^b^	8.65 ± 0.26^ab^	12.45 ± 1.40^a^	12.35 ± 3.45^a^	*
Platelet count (×10^3^/μl)	158.10 ± 8.83^a^	151.75 ± 11.62^ab^	148.80 ± 6.40^ab^	115.50 ± 29.50^b^	NS
Mean platelet volume (fl)	6.90 ± 0.12	7.00 ± 0.11	6.91 ± 0.07	6.75 ± 0.25	NS
Platelet distribution width (fl)	10.78 ± 0.16	10.98 ± 0.17	10.84 ± 0.11	10.95 ± 0.35	NS
Plateletcrit (%)	0.11 ± 0.01	0.11 ± 0.01	0.10 ± 0.01	0.08 ± 0.03	NS

Hemogram values expressed as mean ± SE (Standard error) for each technique. Means followed by different superscripts (^a^, ^b^, ^c^) within the same row are significantly different at (*P* < 0.05). Two means that are not follow by the same letter (a, b, or c) are significantly different (*P* < 0.05). Two means followed with the same letter implies that they are not significantly different. Sig.: represents statistical significance. * *P* < 0.05**.** NS = non- significant. MCHC = mean corpuscular hemoglobin concentration

Compared to the control non-infected group, there was a significant decrease in the mean levels of RBCs, HCT%, Hb and MCHC in the samples derived from *Babesia* sp.-infected cattle, regardless of the method of detection (blood film, cELISA, PCR, or all of them combined). In contrast, MCV values were found increased in the infected group compared to the control group regardless of the method of detection. The result suggests that *Babesia* caused macrocytic hypochromic anemia in the persistently infected animals. In addition, the *Babesia* sp.-infected animals had, on average, significant reduced platelet counts (Table 4).

In infected groups of cattle with *Babesia* sp., as detected by blood film, total leukocyte count (WBCs) and granulocytic counts (neutrophils, eosinophils and basophils) were markedly increased compared to the control non-infected group. On the other hand, there was a decrease in lymphocytic count in infected groups with *Babesia* sp. detected by PCR and all other diagnostic methods (Blood film + cELISA + PCR) compared to the control group. Additionally, no significant changes in the count of MID cell were recorded in all groups (Table 4).

Interestingly, no significant differences were found in any of these blood parameters among buffaloes naturally infected with *Babesia* sp. and the control non-infected group (Table 5).

## Discussion

Bovine and buffalo babesiosis remains neglected in Egypt, since its incidence and overall impact remains poorly assessed, and no preventive control measures, including the use of attenuated vaccines, are applied currently.

Our direct blood film microscopic examination data is generally consistent with previous reports of the presence of bovine babesiosis in distinct regions in Egypt, such as 12.5 % in Cairo [5], 11.1 % in Gharbia Governorate [30], 13 % in Giza Governorate [31], 13 % in Port Said Governorate [32]. Abd-El-Gawad [33] and Mazyad and Khalaf [34] reported 9.9 % in Beni-Suef Governorate and 8.1 % in North Sinai Governorate.

Competitive ELISA is an adequate serological tool for the epidemiological surveillance of the spread of bovine babesiosis, as it can be easily standardized, is less subjective and less time-consuming than the traditionally used IFAT. In addition, cELISA has the potential to display higher specificity than an indirect ELISA [35]. In the current study, the cELISA assays detected a higher number of infected animals than the PCR assays; this could be explained by differences in the timing of the parasite presence and the antibody responses in the infected animals as well as the stage of infections [36, 37]. Consistently, similar studies such as those by Ibrahim *et al.* [38], Silva *et al.* [39] in Portugal, Terkawi *et al* [40] in Thailand and Dominguez *et al* [35] in Argentina, recorded higher babesiosis infection rates when using ELISA assays compared to that using nPCR methods. Yet, the combination of serologic testing and PCR is considered to offer the greatest sensitivity for babesiosis diagnosis especially through the sub-clinical phase.

Results in the current study, suggests that *B. bigemina* infections in bovines are more frequent than *B. bovis*, which is consistent with previous surveys performed in other locations in Africa. Furthermore, a similar finding has been observed by Ibrahim *et al* [38], who reported that *B. bigemina* is more prevalent than *B. bovis* in Egypt. It is possible that *B. bigemina* is more prevalent than *B. bovis* due likely in part to the pattern of geographic distribution of the *R. annulatus* tick vectors [41], added to the fact that the prevalence of *B. bigemina* is higher than *B. bovis* in this vector in Egypt [5]. We also found a significant number of animals that were co-infected with *B. bovis* and *B. bigemina*. This is not surprising, since similar co-infections are commonly reported in endemic areas, and is in part justified by both parasites sharing the same tick vectors. However, transmission of these two parasites by *Rhiphicephalus* sp. ticks is by different mechanisms. While *B. bovis* is transmitted by the larval stage of the ticks, *B. bigemina* is transmitted by the nymph tick stages. Some previous reports suggested that priming the bovine immune system by infection with *B. bigemina* could protect animals of future co-infections with *B. bovis*. Because ticks can also be co-infected, it can be speculated that co-infected animals receive both parasites simultaneously, before they were able to mount cross-protective immune responses that would prevent super-infections [42].

In this study we developed novel snPCR and nPCR methods based on the recently described *B. bovis rra* gene [26] and *B. bigemina rap-1c* gene [25], respectively*.* Sequence comparisons demonstrated that these two genes are highly conserved among distinct strains worldwide, and, importantly, as these two genes are species-specific, they may be better candidates for PCR-based diagnosis of bovine babesiosis. In addition, these two genes generate transcripts in the blood stages of the parasites in a tightly regulated fashion, yet they appear to be expressed in minute amounts [25, 26]. This feature, at least in theory, minimizes the possibility for sequence variation due to immune pressure, and further supports the use of these two genes as ideal targets for developing PCR reactions with diagnostic applications. Additionally, comparisons performed using the new tests with the previously developed nPCR methods, show similar performances in terms of sensitivity and specificity; however, the PCR products are more clearly visualized upon performing both novel PCR tests. Furthermore, searches of the buffalo and bovine genomes demonstrate lack of significant identity of the nPCR primers and full target genes with any bovine or buffalo genes (data not shown), thus diminishing the risk of potential interferences and/or non-specific PCR amplifications. The PCR results obtained regarding the presence of *B. bovis* in cattle in our study are similar to those of Adam *et al* [5], Rania [43] and El-Fayomi *et al* [31] who reported 20 %, 25.33 % and 23 % infection rates in Egypt, respectively. Also, Silva *et al* [39] in Portugal, Chaudry *et al* [44] in Pakistan and Longzheng *et al* [45] in Philippines used nPCR methods for diagnosis of *Babesia* sp. in cattle, and found infection rates of 78.5 %, 29 % and 18.8 %, respectively. However, percentages of *B. bovis* infected buffaloes in our dataset, as detected by PCR, were higher than previously published for samples analyzed from other Egyptian herds. These variations in the prevalence rates are expectable due to geographic diversity and other environmental factors that can influence the population densities of the tick vectors [46]. It is well known that increases in temperature and changes in environmental humidity may influence the migration of vectors into new areas, and/or allow significant development of parasites [31, 47].

Interestingly, despite the identification of buffaloes infected by *B. bigemina* using the cELISA serological method and the presence of infected erythrocytes in the stained slides, *B. bigemina* infections in buffaloes were not detected by the PCR method. On one hand, these observations suggest that the parasites found in the direct microscopy analysis of the buffalo samples are likely *B. bovis*. On the other hand, these data also suggests that the numbers of *B. bigemina* parasites circulating in infected buffaloes may be below the level of detection of the PCR methods, or alternatively, that these *B. bigemina* infected buffaloes successfully cleared the parasites at the time of the samplings. Consistently, and in contrast to *B. bovis*, *B. bigemina* parasites do not sequester in capillaries, and bovines are able to clear *B. bigemina* faster than *B. bovis.* Also, these data is in agreement with a recent survey performed in Egypt using a PCR method which failed to identify a single *B. bigemina* infected buffalo using PCR assays in a sample of 50 animals from different locations [11]. It follows that *B. bigemina* infections in buffaloes might perhaps be more accurately performed using serological, rather than molecular methods, and that, in contrast to *B. bovis*, buffaloes might not be highly significant as potential reservoirs of *B. bigemina* in Egypt.

Hematological findings in this study revealed a marked decrease in the mean levels of RBCs, HCT%, Hb and MCHC and increase in MCV values in cattle infected with *B. bovis* and/or *B. bigemina* regardless of the method of detection (Blood film, cELISA, and PCR) used in this study, and even assuming that these parameters can be affected in these animals due to other unknown reasons different from *Babesia* infections. However, this is an unlikely occurrence, since the non-infected population consistently shows hematological values that are in the normal range. Collectively, these results revealed that *B. bovis* and/or *B. bigemina* likely caused macrocytic hypochromic anemia, indicative of severe intravascular hemolysis of red blood cells in cattle affected with persistent babesiosis [38, 48, 49]. These may be due to the fact that although *Babesia* sp*.* may cause direct damage on some erythrocytes, immune-mediated injury of parasite may be more important in the pathogenesis of anemia [7]. Yet, the increase in erythrophagocytosis by activated macrophages [50, 51] and the production of anti-erythrocyte antibodies [8] may also contribute to the development of anemia.

The reduced platelet counts (thrombocytopenia) were noted in *B. bovis* and/or *B. bigemina* infected cattle with acute, chronic and sub-acute stages of babesiosis, as detected by all the diagnostic methods used in this study (blood film, cELISA, PCR, Blood film + cELISA + PCR). Thrombocytopenia is usually present with protozoal infections of erythrocytes. The increased platelet consumption may be attributed to an immune-mediated process, or it can result from intravascular disseminated coagulation in severe disorders [52, 53]. However, thrombocytopenia is more often associated with increased phagocytosis of platelets in response to antibodies on their surfaces and/or because of macrophage activation by inflammatory cytokines such as macrophage colony-stimulating factor (M-CSF) and Interferon gamma (IFN-γ) [54]. Consistently, splenomegaly, which is concurrent with many parasitic tick-borne diseases, may also be associated with increased platelet sequestration and destruction by splenic macrophages [9].

The increase in total leukocyte count (WBCs) and granulocytic counts found in cattle infected with *B. bovis* and/or *B. bigemina* as detected by blood film (acute stage of babesiosis) compared to control group together with decrease in lymphocyte counts in the groups infected with *Babesia,* when compared to the control group are in agreement with results previously reported by Court *et al.* [51]. These authors also proposes that the significant increase in neutrophils of cattle infected with *B. bovis* could be attributed to their role as active mediators in the innate immune response. Taken together the observations derived from the hematological studies collected in this study can help to assess the overall impact of babesiosis on the health of the cattle and buffalo populations exposed to babesiosis in Egypt.

Remarkably, no such significant hematological changes were recorded in buffaloes naturally infected with *B. bovis* and/or *B. bigemina* in acute, chronic and sub-acute stages of babesiosis. Similar results were reported by Mahmmod [55], who recorded that buffaloes infected with *B. bovis* have less significant hematological changes than cattle infected with the same parasite. These observations, added to the inability of the nPCR to detect *B. bigemina* infections in buffaloes are consistent with the perceived decreased susceptibility of buffalos to *Babesia* infections.

## Conclusions

We estimated high presence of babesiosis caused by *B. bovis* and *B. bigemina* in a diverse population of bovines in Egypt using distinct diagnostic classic, serological and novel molecular approaches. The cELISA data suggests that both parasites are able to circulate among both, bovines and buffalo herds in Egypt, perhaps due to sharing vectors competent for the transmission of both *B. bovis* and *B. bigemina* parasites. Hematological profiles in bovines indicate that infected populations present hematological disorders compatible with intravascular hemolysis and thrombocytopenia regardless of the stage of the disease. However, hematological changes were less evident in buffaloes naturally infected with *Babesia sp.* when compared to cattle.

## Abbreviations

*B. bovis*: *Babesia bovis*
*B. bigemina*: *Babesia bigemina*
EDTA: Ethylene diamine tetra-acetic acid
IFAT: Indirect fluorescent antibody test
ELISA: Enzyme-linked immune-sorbent assay
cELISA: competitive ELISA
gDNA: Genomic DNA
PCR: Polymerase chain reaction
nPCR: Nested PCR
snPCR: Semi-nested PCR
rap-1c: rhoptry associated protein-1c
rra: rap-1 related antigen
fg: femtograms
O.D.: Optical density
RBC: Red blood cell count
HCT: Hematocrite
Hb: Hemoglobin
MCV: Mean corpuscular volume
MCH: Mean corpuscular hemoglobin
MCHC: Mean corpuscular hemoglobin concentration
RDWA: Red blood cell distribution width absolute
RDW%: Red cell distribution width percentage
WBC: White blood cell count
MID: Mid cells
PLT: Platelet count
MPV: Mean platelet volume
PDW: Platelet distribution width
PCT: Plateletcrit
LPCR: Large platelet concentration ratio
ME: Microscopy examination
PPE: Percentage of parasitized erythrocytes
